# Nicotine Elicits Prolonged Calcium Signaling along Ventral Hippocampal Axons

**DOI:** 10.1371/journal.pone.0082719

**Published:** 2013-12-05

**Authors:** Chongbo Zhong, David A. Talmage, Lorna W. Role

**Affiliations:** 1 Department of Neurobiology and Behavior, State University of New York at Stony Brook, Stony Brook, New York, United States of America; 2 Center for Nervous System Disorder, State University of New York at Stony Brook, Stony Brook, New York, United States of America; 3 Department of Pharmacological Science, State University of New York at Stony Brook, Stony Brook, New York, United States of America; 4 Neuroscience Institute, State University of New York at Stony Brook, Stony Brook, New York, United States of America; The University of Tokyo, Japan

## Abstract

Presynaptic nicotinic acetylcholine receptors (nAChRs) have long been implicated in the modulation of CNS circuits. We previously reported that brief exposure to low concentrations of nicotine induced sustained potentiation of glutamatergic transmission at ventral hippocampal (vHipp)-striatal synapses. Here, we exploited nAChR subtype-selective antagonists and agonists and α7*nAChR knockout mutant mice (α7-/-) to elucidate the signaling mechanisms underlying nAChR-mediated modulation of synaptic transmission. Using a combination of micro-slices culture from WT and α7-/-mice, calcium imaging, and immuno-histochemical techniques, we found that nicotine elicits localized and oscillatory increases in intracellular Ca^2+^ along vHipp axons that persists for up to 30 minutes. The sustained phase of the nicotine-induced Ca^2+^ response was blocked by α-BgTx but not by DHβE and was mimicked by α7*nAChR agonists but not by non-α7*nAChR agonists. In vHipp slices from α7-/- mice, nicotine elicited only transient increases of axonal Ca^2+^ signals and did not activate CaMKII. The sustained phase of the nicotine-induced Ca^2+^ response required localized activation of CaMKII, phospholipase C, and IP_3_ receptor mediated Ca^2+^-induced Ca^2+^ release (CICR). In conclusion, activation of presynaptic nAChRs by nicotine elicits Ca^2+^ influx into the presynaptic axons, the sustained phase of the nicotine-induced Ca^2+^ response requires that axonal α7*nAChR activate a downstream signaling network in the vHipp axons.

## Introduction

Neuronal nicotinic acetylcholine receptors (nAChRs) influence the excitability of circuits that underlie fundamental aspects of behaviors related to memory, motivation and mood [1-6]. Dysregulation of central nicotinic signaling is linked to devastating neurodegenerative and neuropsychiatric disorders including schizophrenia, Alzheimer’s disease, depression, anxiety and drug addiction [7-12]. Neuronal nAChRs have been proposed as potential therapeutic targets for cognitive dysfunctions associated with Alzheimer’s disease and schizophrenia [13–15].

Functional nAChRs exist as heteromeric pentamers, comprised of combinations of α (α2-6) and β (β2-4) subunits, or homomeric pentamers, comprised of α (α7–9) subunits [3,16,17]. The most abundant nAChRs in the brain are α7-containing (α7*) and α4β2-containing (α4β2)* subtypes with distinct biophysical and pharmacological properties [18]. Previous studies have shown that (α4β2)* and α7* nAChRs are localized in various cellular domains, including cell bodies, pre-synaptic terminals, post- and peri-synaptic sites [19–21]. Electrophysiological, immunochemical and pharmacological evidence support the presence of (α4β2)* and α7*nAChRs on presynaptic glutamatergic axon terminals, where they modulate the strength of glutamatergic neurotransmission [19,22-25]. Modulation of the release of neurotransmitters (including glutamate, GABA, ACh, and dopamine) by activation of presynaptic nAChRs is the most prevalent mechanism of nicotinic facilitation of synaptic transmission in the CNS [22,23,26,27]. Although nicotinic modulation of circuit excitability by activation of presynaptic nAChRs is critical to CNS function [28-32], the mechanisms by which nAChR activation leads to long-term changes in presynaptic function are not known.

We previously reported that brief exposure to low concentrations of nicotine induced sustained (>30min) potentiation of glutamatergic transmission at ventral hippocampal-striatal synapses [33]. Here, we have exploited nAChR subtype-selective antagonists and agonists and α7*nAChRs knockout mutant mice to elucidate the presynaptic cellular mechanisms underlying the nAChR-mediated sustained synaptic potentiation. 

## Materials and Methods

### vHipp micro-slices cultures and vHipp-nAcc synaptic co-cultures

All animal experiments were carried out in accordance with the National Institutes of Health Guide for the Care and Use of Laboratory Animals (NIH Publications No. 80-23, revised 2012) and studies were approved by Institutional Animal Care and Use for Research Committees at Stony Brook University (#1618 and #1792). The cultures were prepared as described previously [33]. Briefly, for **vHipp micro-slices cultures**, the region of ventral CA1 and subiculum from a single WT or α7 -/- mouse (postnatal day 0-3, P0-P3) were dissected, further sliced into 150×150 μm pieces, and then plated onto poly-D-lysine/laminin-coated glass coverslips (BD Sciences, Bedford, MA) in a minimal volume (50 μl) of culture media (Neurobasal, 2% B-27 (GIBCO, Grand Island, NY) and 20 ng/ml brain-derived neurotrophic factor (R&D Systems, Minneapolis, MN)) to facilitate attachment. After the microslices settled (1-3 hours at 37°C), 100 μl of culture media was added. For **vHipp-nAcc synaptic co-cultures**, nucleus accumbens (nAcc) neurons (ED18 – P1) from WT mice (C57BL/6J) were dispersed with 0.25% trypsin (GIBCO, Grand Island, NY) for 15 min at 37°C, followed by gentle trituration in culture media. Dispersed nAcc neurons were added to the vHipp microslices plated the prior day at 0.25 ml/coverslip. Cultures were maintained in a humidified 37°C, 5% CO_2_ incubator. To ensure the projections we analyzed were from vHipp, in some experiments, the vHipp microslices were prepared from GFP-reporter transgenic mice. With this co-culture system, we have found that projections from vHipp microslices can make glutamatergic synapses with dispersed nAcc neurons as presynaptic axons [33]. In this study, we used vHipp micro-slices culture alone as presynaptic axons for most of the calcium imaging and immunostaining experiments.

### Immunostaining and Fluorescent Visualization

For standard immuno-detection, cultures were fixed in 4% paraformaldehyde/4% sucrose /PBS (20 min, Room temperature), permeabilized with 0.25% Triton X-100/ PBS (5 min, RT), blocked with 10% normal donkey serum in PBS (30 min, RT), and then incubated in primary antibodies overnight at 4°C. The following primary antibodies were used: anti- nAChRs (α_4_ subunits) (1:500, Sigma-Aldrich), anti-vesicular glutamate transporter 1 (1:250, Synaptic Systems, Goettingen, Germany), anti-GAD65 (1:100, Developmental Studies Hybridoma Bank, San Diego, CA, USA), anti-Pan**-**Axonal Neurofilament Marker (1:1000, Santa Cruz Biotechnology, Inc. Santa Cruz, CA, USA), anti-CaMKII (1:500, Santa Cruz), anti-phospho-CaMKII (1:500, Santa Cruz), anti-MAP2 (1:1000, Santa Cruz), anti-GFAP (1:1000, Santa Cruz). Cultures were washed and incubated in secondary antibodies conjugated to Alexa 488 (1:500; Invitrogen) or Alexa 594 (1:500; Invitrogen) for 1 h at RT. Slips were mounted using VectaShield (with DAPi, Vector Laboratories), and images were captured using a microscope (Axio Imager A1; Carl Zeiss, Inc.) equipped with Plan-Apochromat objectives (20× with 0.8 NA or 63× oil with 1.4 NA), a CCD camera (Hamamatsu), and Metamorph software (Version 7.1, Molecular Devices).

To label surface α7*nAChRs, cultures were incubated in αBgTx conjugated to Alexa 594 (1:1000; Molecular Probe) for 30 min at 37°C prior to fixation. Number of surface α7*nAChR clusters were measured along vGluT1-positive processes (≥10 μm from micro-slice) using Metamorph software. The lengths of axonal projections were also measured by tracing vGluT1-positive projections in Metamorph. Control cultures in each experiment were used to define the threshold for clusters at 50% maximum intensity and greater than or equal to four contiguous pixels [34]. To determine nonspecific binding, cultures were treated with 1 μM nicotine before labeling. For each experiment, nonspecific labeling was ≤12% and was subtracted from all counts. Linescans with widths of 10 contiguous pixels were obtained using Metamorph software.

### FM1-43 based imaging and analysis

Activity-dependent FM1-43 dye has been used to detect functional presynaptic boutons [35]. After 5-7 days in vitro, vHipp microslices were loaded with 10 μM FM1-43 (Molecular Probes, Eugene, OR) in 56 mM K^+^ ACSF for 90 s, external dye was washed away in Ca^2+^ free HEPES buffered saline (HBS, 135 mM NaCl, 5 mM KCl, 1 mM MgCl_2_, 10 mM HEPES, 10 mM glucose, pH 7.4) containing ADVASEP-7 (0.1 mM, Sigma) to scavenge membrane-bound FM1-43 for 10–15 min, challenged with 56 mM K^+^ ACSF without FM1-43 for 120 s (destaining), restained with FM1-43 by using the same condition, and washed again with Ca^2+^ free HBS for 10–15 min. The cultures were subsequently maintained in an imaging chamber (Live Imaging Services, Olten Switzerland; containing 1 ml fresh normal HBS) mounted on a Olympus IX81 DSU (spinning disk confocal) microscope (Olympus America Inc., Center Valley, PA) under continuous perfusion (1 ml/min) with HBS containing 2 μM tetrodotoxin (TTX, Tocris), 10 μM bicuculline (Tocris), 50 μM D-AP-5 (Tocris), 20 μM CNQX (Tocris) and 10 μM LY341495 (Tocris). Fluorescence images of vHipp axons were collected by a Plan-Apochromat objective (60× oil with 1.4 NA, excitation 488 nm, emission 530 nm) and captured with a CCD camera (Hamamatsu) every 10 s for 10 min. Image acquisition was performed using the Slidebook software (Version 5, Olympus). After 1 minute of baseline data collection, nicotine (1μM) was applied by rapid perfusion (2 ml/min) for 1 minute to confirm that nicotine can induce destaining of FM1-43 dye–filled vesicles. The total amount of releasable fluorescence at each synaptic bouton was calculated from the difference between fluorescence intensity after staining and after destaining (Δ*F* = *F*_staining_-*F*_destaining_). Fraction of fluorescence intensity decrease after nicotine (F_decrease_%=Δ*F/F*_staining_) were calculated and analyzed. Number and size of FM1-43 positive puncta were measured and compared along vHipp axons from WT *vs.* α7 -/- mouseusing Metamorph software. The lengths of axonal projections were also measured by tracing vHipp projections in Metamorph. 

### Calcium Imaging

After 5-7 days *in vitro*, vHipp microslices were rinsed with HBS (135 mM NaCl, 5 mM KCl, 1 mM MgCl_2_, 2 mM CaCl_2_, 10 mM HEPES, 10 mM glucose pH7.4), loaded with 5 µM Fluo-4 Ca^2+^ binding dye (AM ester, Molecular Probes) and 0.02% Pluronic® F-127 (Molecular Probes) in HBS for 30 min at 37°C and 5% CO_2_. The Fluo-4 solution then was replaced with HBS and the cultures were allowed to recover for at least 30 min at 37°C / 5% CO_2_. The cultures were subsequently maintained in the same imaging chamber and same conditions as described in **FM1-43 based imaging and analysis section**. Fluo-4 fluorescence images of axonal projections from the vHipp micro-slices were collected by a Plan-Apochromat objective (60× oil with 1.4 NA, excitation 488 nm, emission 530 nm) and captured with a CCD camera (Hamamatsu) every 10 s for 30 min. Image acquisition was performed using the Slidebook software (Version 5, Olympus). After 5 minute of baseline data collection, nicotine (1μM) was applied by rapid perfusion (2 ml/min) for 1 minute. The contributions of different subtypes of nAChRs were assessed by including either α-Bungarotoxin (αBgTx, 100 nM, Tocris) to block α7*nAChR, or dihydro-β-erythroidinehydrobromide (DHβE, 1μM, Tocris) to block non-α7*nAChR, in perfusion media, or by activating nAChRs with 1 μM PNU282987 (Tocris), a α7*nAChR specific agonist or 10 μM RJR-2403 (Tocris), a non-α7*nAChR specific agonist.

All frames of the raw fluo-4 fluorescence images were saved as slidebook files and then exported as a series of TIF format images that are then imported to MetaMorph software (Version 7.1, Molecular Devices) and transferred as Z-stack images for further analyses. After setting the threshold of the fluo-4 fluorescence, the integrated intensity of the axonal signals before and after nicotine application was collected and calculated. Fluorescence data are displayed as a normalized integrated intensity: [Δ*F*/F_*0*_= *(*F - F_*0*_)/F_*0*_], where *F*_*0*_ is the background-corrected pre-nicotine fluorescence. Data were analyzed further using Excel software. To examine the distribution of fluo-4 fluorescence signals, the data were assessed in boxplots with Statview software where the boxes include data points between the twenty-fifth percentile (bottom line) and the seventy-fifth percentile (top line).The middle line indicates the fiftieth percentile (median). Vertical lines mark the fifth and ninety-fifth percentiles. If the data were found to be normally distributed statistically significant differences were evaluated by ANOVA with a *post hoc* test for multiple comparisons and group means with unequal sample size. Other data were analyzed using nonparametric methods (Kolmogorov–Smirnovtest).

## Results

### Development of gene chimeric co-cultures for analysis of pre vs. post synaptic contributions to synaptic plasticity

We developed a specialized preparation of hippocampal–striatalcircuits *in vitro* to examine the cellular signaling mechanisms by which presynaptic nAChRs modulate synaptic transmission. Ventral hippocampal and subicular regions (vHipp) were extirpated and micro-slices were plated in minimal volume and allowed to spread before the addition of dispersed target neurons from the nucleus accumbens shell. With this co-culture preparation, we previously demonstrated that nicotine elicits a sustained (>30min) potentiation of glutamatergic transmission via activation of α7*nAChRs [33].

**Figure 1:**
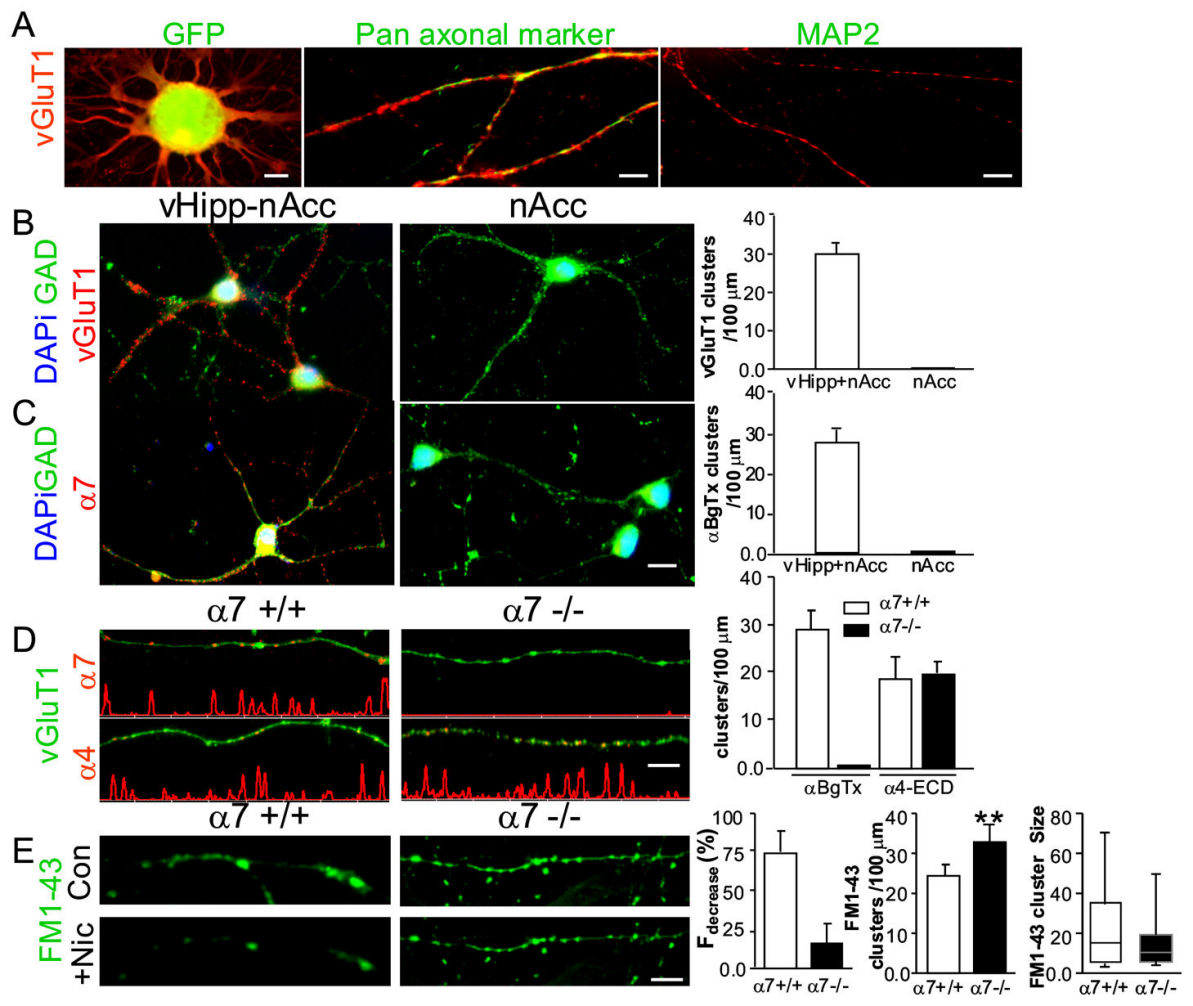
Gene-chimeric co-culture of ventral hippocampus with nucleus accumbens. **A**: Cultures of vHipp microslices from GFP reporter transgenic mice or WT mice (5-7 days *in* *vitro*) were fixed, permeabilized, and stained with antibodies recognizing vGluT1 (red) and a panaxonal marker (SMI312, green) or a dendritic marker (MAP2, green). Microslices of ventral hippocampus from GFP reporter transgenic mice extend processes (**A left**, GFP that are labeled by vGluT1 (red), scale bar: 50μm). These glutamatergic projections are co-labeled by SMI312 (**A middle**, green, scale bar: 10μm), but not by MAP2 (**A right**, green, scale bar: 10μm). **B**, **C**: Cultures of dispersed nucleus accumbens medium spiny neurons with (**B, C Left**) or without (**B, C middle**) vHipp microslices were fixed, permeabilized, and stained with antibodies recognizing GAD65 (green) and vGluT1 (**B**, red) or surface α7*nAChR (**C**, red). Red “clusters” of vGluT1 staining can be found on dispersed nAcc GABAergic neurons with (**B left, right**, 30+/-3.8 per 100 μm fibers) but not without (**B middle**) the co-culture of vHipp microslices. For labeling surface α7*nAChR, the cultures were incubated with αBgTx–Alexa 594 for 45 mins prior to fixation. Red “clusters” of surface α7*nAChR are only seen in cultures that include vHipp microslices (**C left, right**, 28+/-3.2 per 100 μm fibers), dispersed GABAergic neurons from nAcc alone are devoid of αBgTx staining (**C middle**). Scale bar: 10 μm. For quantification, only the “clusters” of vGLuT1 (**B left**) or surface α7*nAChR (**C left**) along the neurites of the green GABAergic neurons were counted. At least 3000 μm lengths of neurites for each group from three independent experiments were collected and quantified. **D**: Cultures of vHipp microslices from WT or α7 -/- mice were labeled for surface α7*nAChR with αBgTx–Alexa 594 or labeled for surface α4*nAChR with antibody recognizing the ECD (extracellular domain) of α4 subunits. Representative micrographs of WT (α7+/+, **D, left**) and α7-/- (**D, middle**) vHipp axons (staining with vGluT1, green) are shown above line scans of fluorescence intensity profile for surface α7*nAChR (**D top**, αBgTx-Alexa-594 staining in “clusters”) and α4*nAChR (**D bottom**, α4-ECD staining in “clusters”). Scale bar: 10μm. Surface α7*nAChR and α4*nAChR clusters along axons from WT (α7+/+) and α7-/- vHipp microslices were quantified (**D right**). Surface α7*nAChR clusters were found along WT (α7+/+) vHipp axons but not α7-/- vHipp axons (27.6 ± 3.9 *vs*. 0 ± 0 clusters/100 µm). α4*nAChR clusters were found along both WT (α7+/+) and α7-/- vHipp axons (17.6 ± 2.8 *vs*. 19.4 ± 3.3 clusters/100 µm). At least 3000 μm axonal lengths for each group from three independent experiments were collected and quantified. **E**: Cultures of vHipp microslices from WT (α7+/+) or α7 -/- mice were loaded with FM1-43. Representative micrographs of WT (α7+/+, **E, left**) and α7-/- (**E, middle**) vHipp axons (loaded with FM1-43, green) before (**E, top**) and after (**E, bottom**) nicotine application are shown. Scale bar: 10μm. Fraction of FM1-43 fluorescence intensity decrease along vHipp axons after nicotine application was quantified (**E right**). Bars show average ± s.e.m. of 4 independent experiments. Nicotine induced FM1-43 destaining were dramatically decreased in vHipp axons from α7 -/- mice (P<0.01, t-Test). The number of FM1-43 positive puncta were compared along vHipp axons from WT *vs*. α7-/- (23.8 ± 2.8 *vs*.32.4 ± 3.6 clusters/100 µm, bars show average ± s.e.m. P<0.01, t-Test, **E right**). Box plot of pooled data shows that there was no statistically significant difference in the size of FM1-43 positive puncta comparing WT with α7-/- (18.6 ± 5.8 *vs*. 14.4 ± 4.3, P=0.056, t-Test, **E, right**). At least 2000 μm axonal lengths for each group from four independent experiments were collected and quantified.

Immunohistochemical methods were used to further probe the distribution of pre- and post-synaptic markers in gene chimeric co-cultures. Ventral hippocampal microslices (5-7 days *in vitro*) were fixed, permeabilized, and stained with antibodies recognizing vesicular glutamate transporter1 (vGluT1) and a pan-axonal neurofilament marker (SMI 312) or a dendritic marker (MAP2). Projections from micro-slices prepared from GFP-reporter transgenic mice demonstrate vGluT1 staining at multiple sites along the projections that exit the explant and project for at least 50 μm (Figure **1A** **, left**). The vGluT1 positive fibers emerging from vHipp micro-slices are identified as axons by co-labeling with SMI 312 (Figure **1A** **, middle**), and the lack of co-labeling with MAP2 (Figure **1A** **, right**).

Dispersed nAcc medium spiny neurons, maintained in vitro for 5-7 days with or without vHipp micro-slices, were fixed, permeabilized, and stained with antibodies for GAD65 and vGluT1. Clusters of vGluT1 staining were only found when GABAergic nAcc dispersed neurons were co-cultured with vHipp micro-slices (Figure **1B** **, left**, **right**, 30±3.8 per 100 μm fibers) consistent with vGluT1 staining of presynaptic sites along vHipp projections. Vesicular Glutamate Transporter 1 staining was never detected in cultures of dispersed GABAergic nAcc neurons alone (Figure **1B** **, middle, right**). 

To examine the distribution of surface α7*nAChRs, the cultures were incubated with αBgTx conjugated to Alexa 594 prior to fixation. To determine the distribution of αBgTx positive sites relative to GABAergic fibers, the cultures were then stained with antibodies for GAD65 after fixation and permeabilization. Surface clusters of αBgTx-594 were detected along the neurites of GAD65 positive neurons if (and only if) the nAcc neurons were co-cultured with vHipp micro-slices (Figure **1C** **, left, right**, 28±3.2 per 100 μm fibers *vs.* Figure **1C** **, middle, right**).

To verify the expression of specific subtypes of nAChRs (i.e. including α7 *vs.* α4 subunits) along vHipp axons, vHipp microslices from WT (Figure **1D** **Left**) or α7-/- mice (Figure **1D** **middle**) were labeled for surface α7*nAChR with αBgTx–Alexa 594 (Figure **1D** **top**) or labeled for α4*nAChR with antibody recognizing the extracellular domain(ECD) of α4 subunits (Figure **1D** **bottom**). Projections were co-labeled with vGluT1 antibodies. Surface α7*nAChR-containing clusters (i.e. αBgTx positive) were found on projections from WT microslices but not on projections from α7-/- vHipp (27.6 ± 3.9 *vs.* 0 ± 0 clusters/100 µm, Figure **1D** **right**). The α4*nAChR clusters were found on both WT and α7-/- vHipp axons; there was no statistically significant difference in α4*nAChR-containing clusters comparing WT with α7-/- (17.6 ± 2.8 *vs.* 19.4 ± 3.3 clusters/100 µm) (Figure **1D** **right**).

These immunostaining studies indicate that the fibers projecting from the vHipp micro-slices that were vGluT1 positive contacted dispersed GABAergic medium spiny neurons from nAcc and that both α7* and non-α7*nAChRs were found along the vHipp axons and specifically at sites where vHipp projections contact nAcc neurons.

To further test whether axonal nAChRs are related to synaptic transmission machinery, the fluorescent styryl dye FM1-43 was used to directly visualize sites of vesicular release and the effects of nicotine. When we imaged clusters of vesicles (puncta) in WT (Figure **1E** **, left, top**) and α7-/- (Figure **1E** **, middle, top**) vHipp axons, the fluorescence intensities and numbers of puncta were stable for at least 60 min in the absence of stimulation. The α7-/- vHipp axons (32.4 ± 3.6 clusters/100 µm) have more FM1-43 positive puncta than the WT vHipp axons (23.8 ± 2.8 clusters/100 µm). There was no statistically significant difference in the size of FM1-43 positive puncta comparing WT with α7-/- (18.6 ± 5.8 *vs.* 14.4 ± 4.3) (Figure **1E** **, right**). After nicotine (1 μM, 1 min) application, the fluorescence intensity of all puncta along WT axons (Figure **1E** **, left bottom, right**) rapidly diminished by ~75%, reflecting exocytosis of dye from synaptic vesicles. In contrast, fluorescence intensity of FM1-43 puncta decreased by ~25% with nicotine exposure of α7-/- vHipp axons (Figure **1E** **, middle bottom, right**). These data are consistent with the idea that α7*nAChRs are required for maximal nicotine induced neurotransmitter release along vHipp axons. In sum, axons projecting from the vHipp microslices develop functional synapse in co-culture and presynaptic specializations when plated alone, that have associated nAChRs. Nicotine activation of these presynaptic nAChRs increases vesicle fusion and neurotransmitter release and spontaneous synaptic activity (Figure **1** and Ref. [33]).

### A brief application of nicotine elicits sustained changes in intracellular Ca^2+^ along axonal projections from vHipp microslices

In a previous study we demonstrated that activation of presynaptic nAChRs elicited both short and long term enhancement of glutamatergic transmission at vHipp-nAcc synapses [33]. In the current study, we sought to identify the signaling pathway(s) involved in the nicotinic modulation of synaptic transmission. Nicotine can induce focal and global calcium transients in cultured primary neurons and in several cell lines [36–38]. To assess the potential contribution of axonal Ca^2+^ signaling, we used *in vitro* vHipp microslices culture preparations and spinning disk confocal imaging with fluo-4 AM to directly monitor local changes in intracellular Ca^2+^ signals along vHipp axons. 

**Figure 2:**
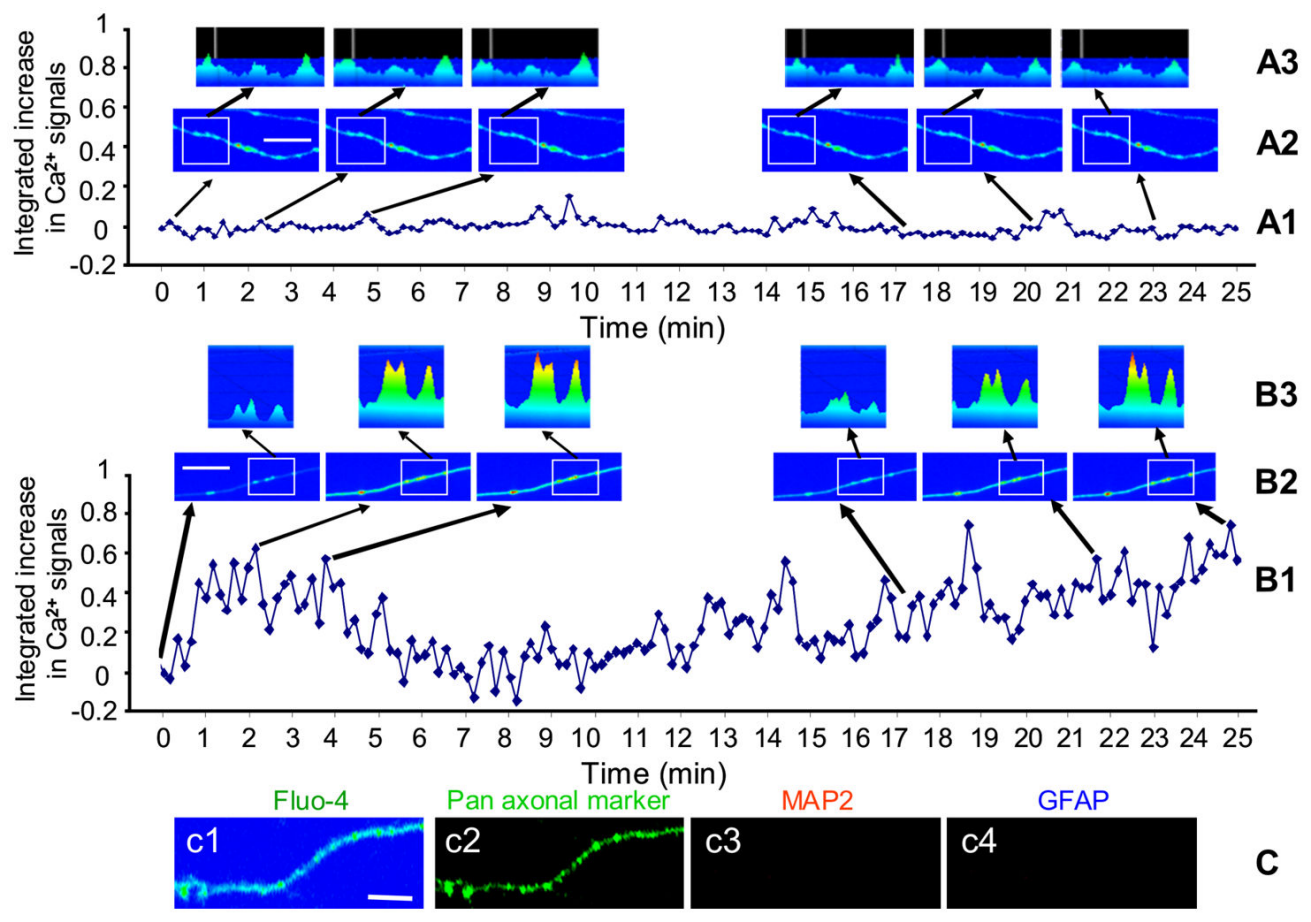
A single application of nicotine induces sustained changes in [Ca^2+^]_i_ along vHipp axons. Spinning disk confocal live Ca^2+^ images from WT vHipp axons were recorded every 10 seconds for 30 min and fluo-4 fluorescence intensities were calculated and quantified as a normalized integrated intensity at each time point. The changes of normalized integrated intensities at one spot of vHipp axons were plotted vs. time. **A1**: Representative plot from a live WT vHipp axon perfused with normal HEPES buffered solution showed that the normal axonal intracellular Ca^2+^ signals oscillated in a random manner with very small amplitude. **A2**: Representative spinning disk confocal fluo-4 images are indicated on pseudo color scale at different time points (0, 2.1’, 4.5’, 17.5’, 20.5’, and 23’). The square area is the region for which fluo-4 intensities were quantified. Scale bar: 5μm. **A3**: Representative spinning disk confocal fluo-4 images are indicated on pseudo color scale in 2D manner from the square area (see A2) at different time points. **B1**: Representative plot from a live WT vHipp axon perfused with nicotine (1μM) for 1 min and then with normal HEPES buffered solution without nicotine. The nicotine application induced both a quick and sustained increase of axonal intracellular calcium signals that oscillated in a random manner with large amplitude. **B2**: Representative spinning disk confocal fluo-4 imagings are indicated on pseudo color scale at different time points after nicotine application (0, 2’, 4’, 17.5’, 22’, and 25’). The square area is the area for which fluo-4 intensities were quantified. Scale bar: 5 μm. **B3**: Representative spinning disk confocal fluo-4 images are indicated on pseudo color scale in 2D manner from the square area (see B2) at different time point. **C**: After recording of Fluo-4/Ca^2+^fluorescence (**c1**), the culture of vHipp microslices were fixed, permeabilized, and stained with antibodies recognizing panaxonal marker (green), MAP2 (red),and GFAP (blue). The vHipp projections previously assessed by calcium imaging and relocated post hoc are co-labeled by panaxonal marker (**c2**), but not by either a dendritic marker MAP2 (**c3**), or by a glia marker GFAP (**c4**), scale bar: 5μm.

Fluo-4 / Ca^2+^ fluorescence images were captured for 500 ms every 10 s over a 30 min recording period before and after nicotine exposure. Analysis of these Fluo-4 / Ca^2+^ fluorescence images revealed a consistent, albeit complex, temporal and spatial pattern of changes in intracellular free Ca^2+^ ([Ca^2+^]_i_). At baseline (assayed for at least 30 minutes without treatments), there were multiple, low amplitude “hot spots” of [Ca^2+^]_i_ at inter hotspot distances of 10 - 30 μm along vHipp axons (Figure **2 A1** **, A2, A3**). Subsequent recordings of Fluo-4 /Ca^2+^signals, during, and for 30-60 minutes after a single 1 min exposure to and subsequent washout of nicotine revealed both a rapid (seconds to several minutes) and a sustained (≥10-30 min) change in the [Ca^2+^]_i_ at multiple presynaptic sites along vHipp axons (Figure **2 B1, B2** **, B3**). Specifically, a typical response to nicotine in WT vHipp axons had the following features: during the nicotine exposure the fluo-4 / Ca^2+^ fluorescence at “hotspots” seen prior to stimulation, and at multiple additional 1-3 μm domains, was greatly increased. The Ca^2+^ signal was not uniform along the axons; rather the changes occurred in discrete foci. Following nicotine washout the amplitude of the Ca^2+^ signal at each of these discrete foci oscillated with a peak to peak phase of approximately 100 seconds above the pre-nicotine baseline. About 10 min post-nicotine washout, the signals had often returned to close to pre-nicotine levels. In axons in which a sustained phase of Ca^2+^ signals were detected, the focal oscillations persisted for another 10 - 30 min. Although the peak amplitude of the Ca^2+^ signal was greatly increased compared with the baseline, the frequency of the oscillations at each hot spot did not appear to change over the imaging sessions. In the following discussion of our results we will refer to changes that occurred during and in the period immediately following the nicotine application and washout as the “initial” response to nicotine, whereas those changes that were quantified 20 - 30 minutes after nicotine washout constitute the “sustained” responses to nicotine.

To verify whether the projections in which we recorded nicotine-induced calcium signaling (Figure **2 c1**) were indeed axons, vHipp microslices were fixed, permeabilized, and stained with antibodies to panaxonal marker (green), MAP2(red), and GFAP (blue). We found that all of the vHipp projections in which we recorded calcium changes in response to nicotine were labeled by panaxonal marker (Figure **2 c2**), but not by either the dendritic marker, MAP2 (Figure **2 c3**), or by the glia marker, GFAP (Figure **2 c4**), consistent with their axonal identity. 

The observation that there is a persistent Ca^2+^ response to a transient exposure to nicotine (and hence, a transient activation of nAChRs) was a highly unexpected outcome. As such, we have probed the time course, pharmacology, and signaling cascades involved in the nicotine-induced Ca^2+^ response in over 100 experiments (Table **1**). First we analyzed nicotine induced calcium signals along 100 vHipp axons (at least 50 μm length for each axon) from WT mice, and found that 76 of those axons showed both the rapid and the sustained calcium signaling responses; 14 only showed the rapid responses and 10 showed no responses at all. Subsequent experiments used genetic and pharmacological methods to dissect the nAChRs subtypes contributing to the initial and sustained phases of the nicotine-induced Ca^2+^ response along presynaptic axons and to explore the potential intracellular signaling mechanisms involved. 

**Table 1:** Number of samples analyzed for Ca^2+^ imaging results.

	Animals (n)	Coverslips (n)	Recording Areas (n)	Axon length (μm)
WT +Nic	15	21	27	~ 5000
α7 -/- +Nic	10	13	15	~ 2500
WT+αBgTx+Nic	9	10	11	~ 2000
WT+DHβE+Nic	8	10	11	~ 2000
WT+PNU282987	7	7	7	~ 2000
WT+RJR-2403	7	7	7	~ 2000
WT+Rya+Nic	7	9	9	~ 2000
WT+Xes-C+Nic	6	8	8	~ 2000
WT+KN93+Nic	6	8	8	~ 2000
WT+AIP+Nic	4	6	6	~ 1500
WT+PP2+Nic	4	6	6	~ 1500
WT+U73122+Nic	4	6	8	~ 1500
Total	83	111	117	26000

Abbreviations: **WT**, Wild Type; **α7-/-**, α7*nAChR knock out mutant; **αBgTx** (α-bungarotoxin), α7*nAChR blocker; **DHβE** (Dihydro-β-erythroidine), non-α7*nAChR blocker; **PNU282987**, α7*nAChR agonist; **RJR-2403**, non-α7*nAChR agonist; **Rya** (ryanodine), ryanodine receptor-sensitive Ca^2+^ stores blocker; **Xes-C** (xestospongin C), IP_3_ receptor-sensitive Ca^2+^ stores blocker; **KN93** and **AIP** (Autocamtide-2-Related Inhibitory Peptide), CaMKII inhibitor; **PP2**, Src tyrosine kinase inhibitor; **U73122**, phospholipase C inhibitor.

### α7*nAChRs is required for the sustained phase of nicotine-induced Ca^2+^ response along vHipp axons

Both α7* and (α4β2)* subtypes of nAChRs contribute to the increase in glutamatergic transmission at vHipp-nAcc synapses. Pretreatment with the α7*nAChR-selective antagonist (αBgTx) eliminated the sustained enhancement of glutamatergic transmission by nicotine [33]. 

**Figure 3:**
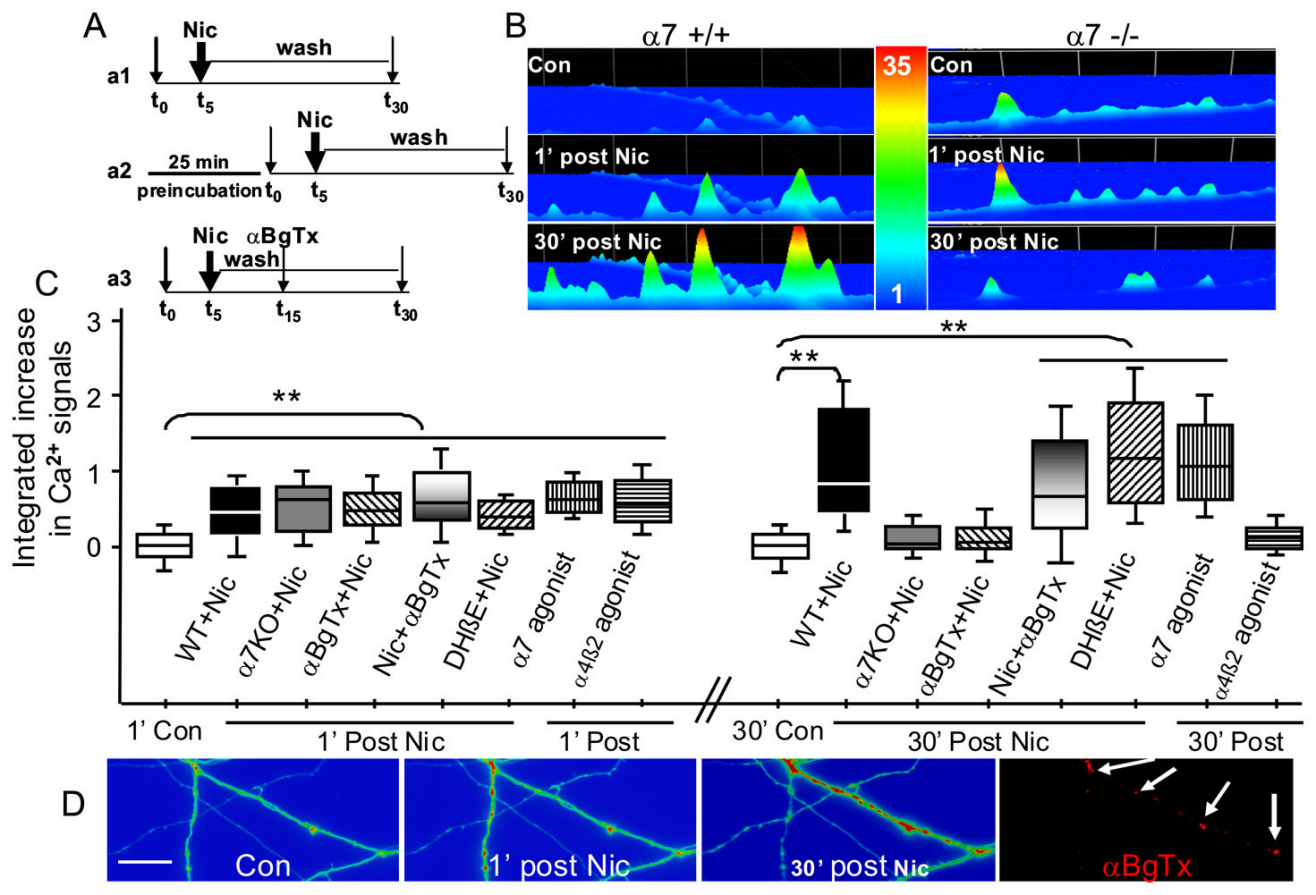
α7*nAChRs participate in nicotine induced sustained changes in intracellular Ca^2+^ in vHipp axons. **A**: Schematic of experimental protocols. a1: Spinning disk confocal images from live WT and/or α7-/- vHipp axons were recorded every 10 seconds for 30 min, including baseline data collection for 5 minutes, and followed by application and washout of nicotine or α7*nAChR or non-α7*nAChR agonist. a2: To dissect out the subtypes of nAChR involved in sustained changes in intracellular Ca^2+^ elicited by nicotine, WT vHipp axons were pre-incubated with an α7*nAChR antagonist (αBgTx) or with a non-α7*nAChR antagonist (DHβE) for 25 minutes respectively, after which the protocol illustrated in part **a1** was followed. a3: WT axonal calcium signals were recorded at baseline followed by nicotine application and wash out. Ten minutes after nicotine application, the α7*nAChR antagonist (αBgTx) was added to address whether inhibition of α7*nAChRs can block Ca^2+^ signaling once the process was initiated. **B**: Representative spinning disk confocal fluo-4 images in pseudo color scale before (Top), 1’ (Middle), and 30’ (Bottom) after nicotine application to WT (**left**) and α7-/- (**right**) vHipp axons. Scale bar: 5μm. **C**: Box plot of pooled data shows that the acute effects of nicotine on fluo-4 fluorescence (***ΔF/F_0_= (F-F_0_)/F_0_***) were comparable for WT (27 recordings in 21 coverslips from 15 mice) and α7-/- (15 recordings in 13 coverslips from 10 mice) vHipp axons. In contrast, the sustained change in intracellular Ca^2+^ seen at 30 min after nicotine treatment of WT vHipp axons was not seen in α7-/- vHipp axons. Preincubation with αBgTx (11 recordings in 10 coverslips from 9 mice) eliminated the sustained phase of nicotine induced intracellular Ca^2+^ response, whereas the DHβE (11 recordings in 10coverslips from 8 mice) did not. RJR-2403 (7 recordings in 7 coverslips from 7 mice) elicited only the acute phase of Ca^2+^ signaling whereas PNU282987 (7 recordings in 7 coverslips from 7 mice) caused sustained Ca^2+^ signaling. Application of αBgTx 10 minutes after nicotine washout had no effect on the subsequent Ca^2+^ response. At least 1500 μm axonal lengths for each group were collected and quantified. **p<0.01. **D**: α7*nAChR clusters are co-localized with the “hot spots” of nicotine-induced sustained Ca^2+^ response along vHipp axons. After recording of nicotine-induced changes of Fluo-4/Ca^2+^ fluorescence (**d1, d2, d3**), the vHipp axons were labeled for surface α7*nAChR with αBgTx–Alexa 594 (**d4**). Relocalization of sites at which nicotine had induced sustained changes in Ca^2+^ signaling along vHipp axons (**white arrow in d3**) revealed that these sites corresponded to sites of positive staining for surface α7*nAChR (**white arrow in d4**). Relocalization of sites where nicotine elicited only acute changes in Ca^2+^ signaling (**orange arrow in d2**) were not labeled by αBgTx–Alexa 594 (**d4**). Scale bar: 10μm.

To further dissect the nAChR subtypes contributing to nicotine-induced Ca^2+^ responses, we compared the effects of nicotine on the Ca^2+^ signal along vHipp axons from WT *vs.* α7-/- mice. The rapid, initial phase of nicotine-induced changes in [Ca^2+^]i were comparable for vHipp axons from WT *vs.* α7-/- mice. In contrast, the sustained phase of the Ca^2+^ responses (i.e. the intracellular Ca^2+^ signals for 20 or 30 min after nicotine washout) that was recorded in roughly 80% of the WT vHipp axons was not detected in comparable assays of any axons from α7-/- mice (Figure **3 B, C**). 

We next compared the effects of antagonists specific for α7* *vs.* (α4β2)*nAChR on nicotine-induced changes in vHipp axonal [Ca^2+^]_i_. Pre-incubation (Figure **3A** **, a2**) with the α7*nAChR selective antagonist αBgTx (100 nM) eliminated the sustained phase of the nicotine-induced Ca^2+^ responses (Figure **3C**). Pre-incubation with the non-α7*nAChR selective antagonist DHβE (1 μM) had no effect on nicotine-induced changes in [Ca^2+^]_i_ along vHipp axons (Figure **3C**). 

Application of PNU282987 (1 μM), a selective agonist for α7*nAChR, elicited ongoing vHipp axonal oscillations in [Ca^2+^]_i_ that were detected for ≥ 30 min. In contrast, application of an agonist that activates all nAChRs except α7*nAChR (RJR-2403, 5μM) elicited only a short term increase of axonal Ca^2+^ (Figure **3C**). 

To clarify whether the activation of α7*nAChRs is required for the initiation or for the maintenance of the sustained Ca^2+^ response, αBgTx was applied 10 minutes after nicotine application (Figure **3A** **, a3**). Under these conditions, the sustained Ca^2+^ response was unaffected (Figure **3C**), consistent with the idea that α7*nAChR activation is only required to initiate the sustained phase of the nicotine-induced Ca^2+^ response.

In several experiments we relocalized the “hot spots” of nicotine-induced Ca^2+^ signal (Figure **3D, d1, d2**) to assess whether we could detect surface α7*nAChR at such sites. Over 90% of all of the relocalized sites at which nicotine had induced sustained changes in Ca^2+^ along vHipp axons corresponded to sites of positive staining for surface α7*nAChRs (Figure **3D, d3, d4**).

Taken together, we find that nicotine-induced increases in [Ca^2+^]_i_ were detected at multiple sites along vHipp axons and, if α7*nAChRs were activated, these signals persisted in oscillatory waves for ≥10-30 min after the removal of agonist. Furthermore, these results indicate that both α7*nAChR and non-α7*nAChR participate in nicotine-induced increases in [Ca^2+^]_i_, but that activation of α7*nAChRs were both necessary and sufficient for inducing the sustained phase of the Ca^2+^ response.

### Calcium-induced calcium release (CICR) is required for the sustained, α7*nAChR mediated changes in [Ca^2+^]_i_ along vHipp axons

**Figure 4:**
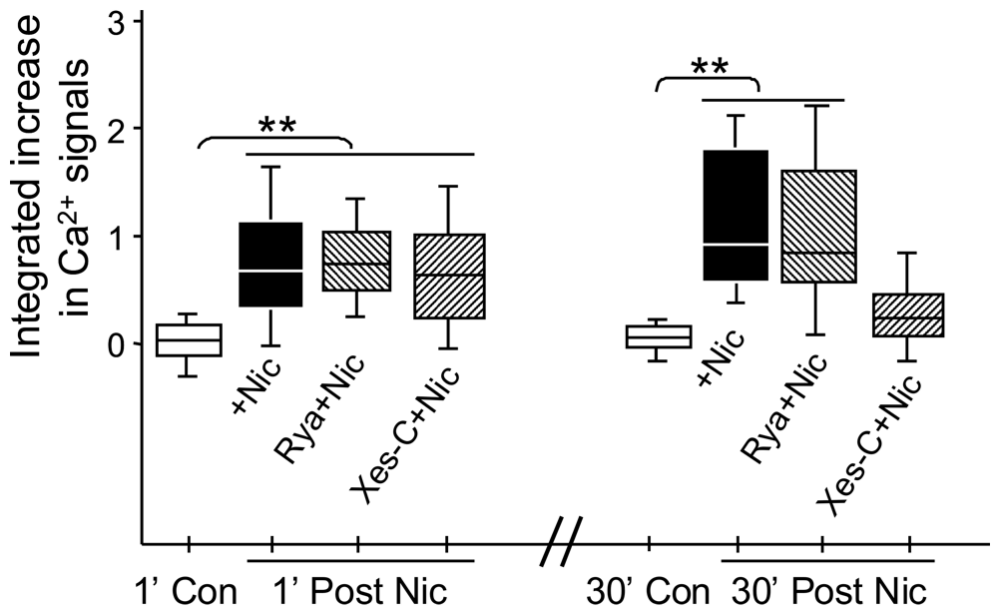
CICR is required for the nicotine induced sustained response in intracellular Ca^2+^ in vHipp axons. Box plot of pooled data shows that the acute effects of nicotine [Δ*F*/ F_*0*_= (F - F_*0*_)/F_*0*_] were not altered by 30 min pre-incubation with a blocker of ryanodine-sensitive endoplasmic reticulum stores, (ryanodine 30 μM, 9 recordings in 9 coverslips from 7 mice) or an inhibitor of IP_3_ receptors, (xestospongin-c 100 nM, 8 recordings in 8 coverslips from 6 mice). In contrast, the sustained change in intracellular Ca^2+^ seen at 30 min after nicotine treatment at WT vHipp axons was still seen in ryanodine treated cultures but not in xestospongin-c treated cultures. At least 1500 μm axonal lengths for each group were collected and quantified. **p< 0.01.

Calcium-induced calcium release (CICR) from intracellular stores has been proposed to contribute to nAChR-mediated modulation of synaptic transmission [27,39,40]. We next used a pharmacological approach to probe the contribution of intracellular Ca^2+^ stores to the nicotine-induced Ca^2+^ response along vHipp axons. Following a 30 min pre-incubation with ryanodine (30 μm), an antagonist that blocks Ca^2+^ release from ryanodine receptor-sensitive Ca^2+^ stores, the intracellular Ca^2+^ responses to nicotine were not changed (Figure **4**). In contrast, pre-incubation with xestospongin C, an inositol-1,4,5-trisphosphate (IP_3_) receptor antagonist that blocks Ca^2+^ release from IP_3_ receptor sensitive Ca^2+^ stores, completely blocked the sustained phase of the nicotine-induced change in [Ca^2+^]_i_. The initial acute phase was unaffected (Figure **4**). Thus, stimulation of a sustained response in [Ca^2+^]_i_ by nicotine requires activation of CICR through IP_3_ receptor activation whereas ryanodine-sensitive Ca^2+^ stores appear to not be necessary. 

### Activation of CaMKII is required for the sustained phase of nicotine-induced changes in [Ca^2+^]_i_ along vHipp axons

**Figure 5:**
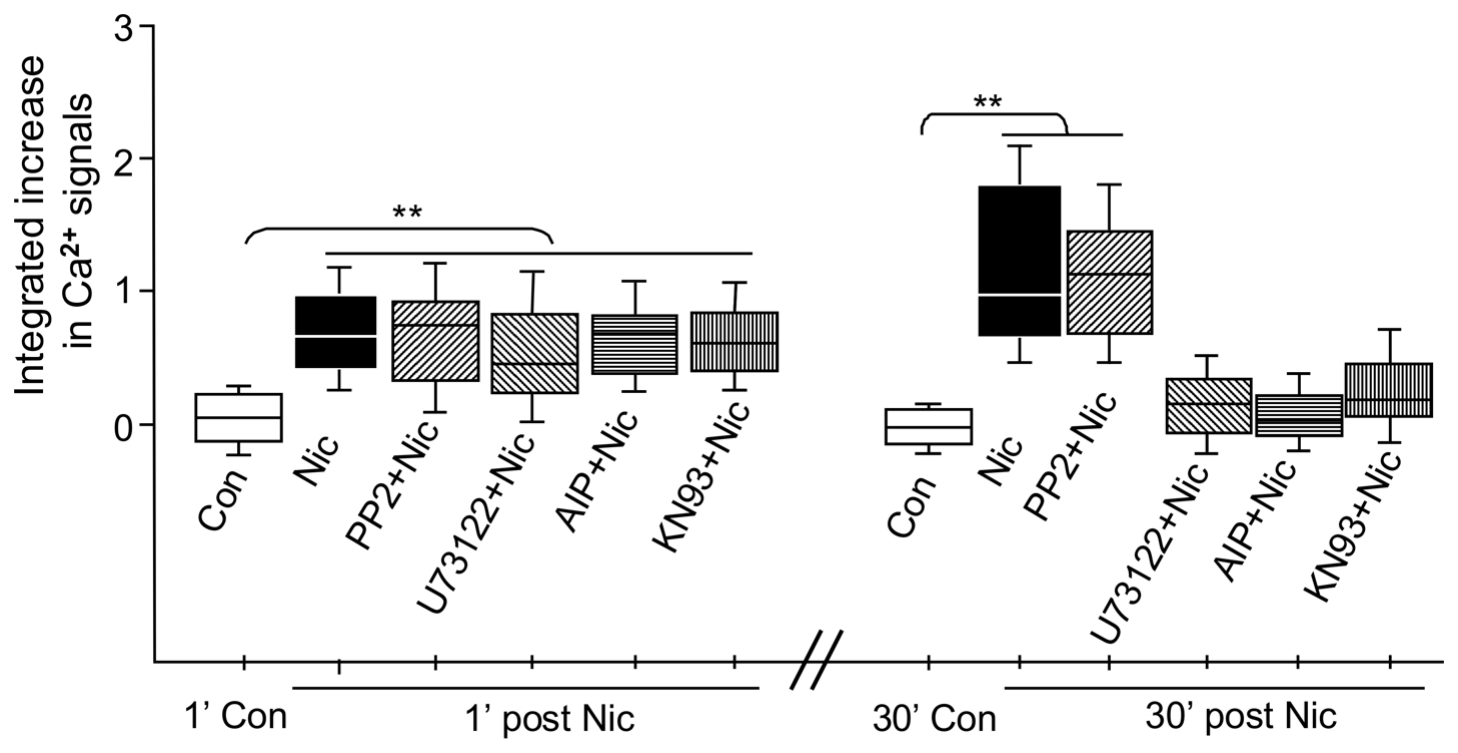
Activation of PLC and CaMKII is required for the sustained, nicotine induced change in intracellular Ca^2+^ in vHipp axons. Box plot of pooled data on nicotine induced Ca^2+^ signaling [Δ*F*/ F_*0*_= (F - F_*0*_)/F_*0*_] after 30 minutes of pre-incubation with inhibitors of CaMKII, (KN93; 5 μM, 8 recordings in 8 coverslips from 6 mice andAutocamtide-2-Related Inhibitory Peptide; AIP, 20 μM, 6 recordings in 6 coverslips from 4 mice). Although CaMKII inhibitors had no effect on the acute phase of Ca^2+^ signaling, the sustained phase was completely blocked by pre-incubation with KN93 or AIP. A 30 min pre-incubation with an inhibitor of phospholipase C (U73122, 10 μM, 8 recordings in 6 coverslips from 4 mice) or Src tyrosine kinase inhibitor (PP2, 20 μM, 6 recordings in 6 coverslips from 4 mice) did not affect the acute phase of nicotine induced Ca^2+^ signaling. In contrast, the sustained phase was blocked by U73122, but not by PP2. At least 1500 μm axonal lengths for each group were collected and quantified. **p<0.01 .

In non-neuronal cells transient increases in intracellular calcium occur in response to α7*nAChR activation [38]. In these examples, α7*nAChR effects on calcium are mediated either by src family tyrosine kinase activation or phospholipase C / IP_3_ signaling. Preincubation of vHipp cultures with the src family kinase inhibitor, PP2, had no effect on the sustained phase of the nicotine induced calcium signaling (Figure **5**). In marked contrast, the general phospholipase C inhibitor, U73122 (10 μM, 30 min) eliminated the sustained phase of the nicotine-induced response in [Ca^2+^]_i_.

Because of the short half-life of IP_3_ [41] and kinetics of the IP_3_ receptor [42], IP_3_ receptor mediated CICR is typically a short lived response. As such, we postulated that some aspect of signaling downstream of the activation of CICR was required for the sustained changes in [Ca^2+^]_i_ after nicotine exposure. We tested whether activation of Ca^2+^/Calmodulin-Dependent Protein Kinases II (CaMKII) might influence IP_3_ receptor mediated CICR. In these studies, vHipp preparations were pre-treated for 30 min with the CaMKII inhibitor KN93 (5 μM) or Autocamtide-2-Related Inhibitory Peptide (AIP, 20 μM) prior to nicotine stimulation. In the presence of KN93 or AIP, nicotine still elicited an acute increase in calcium signal, but the sustained phase of the nicotine induced response in [Ca^2+^]_i_ was blocked (Figure **5**). Together these findings support the proposal that activation of IP_3_ receptor-mediated CICR by CaMKII is an essential component of the sustained, α7*nAChR mediated response in intracellular Ca^2+^.

### A single application of nicotine elicits sustained activation of CaMKII along vHipp axons

**Figure 6:**
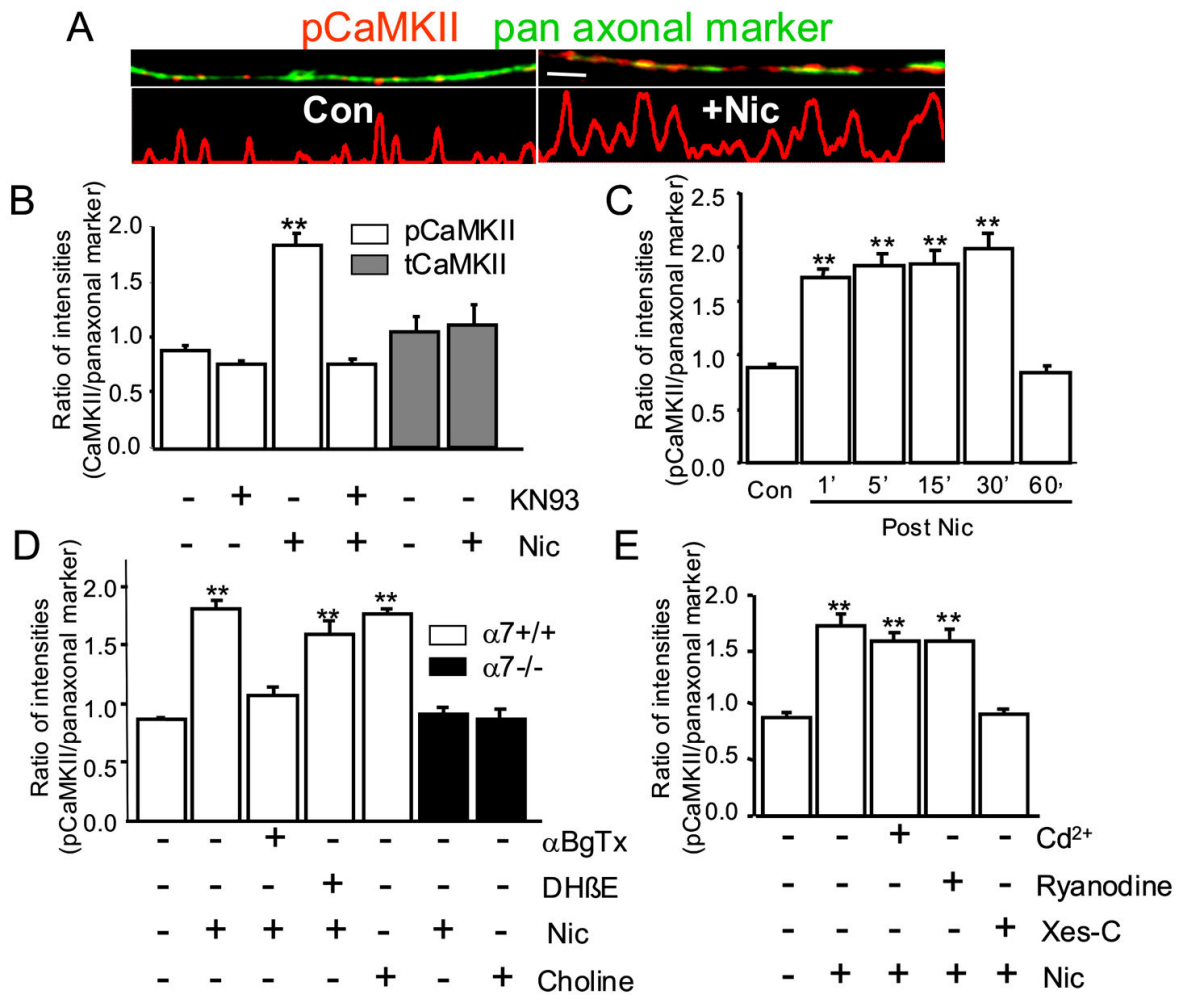
α7*nAChR and CICR are required for sustained, nicotine induced activation of CaMKII along vHipp axons. vHipp microslice cultures from WT mice were fixed after incubation with nicotine, and then permeabilized, and stained with antibodies recognizing phospho-CaMKII (red) and axonal neurofilaments (green). **A**: Representative micrographs of WT vHipp axons are shown above line scans of fluorescence intensity profile for phospho-CaMKII staining; control *vs*. 5 min after nicotine (Scale bar: 10 μm). **B**: phospho-CaMKII immunofluorescent intensities along axons were quantified (as ratio of phospho-CaMKII/pan-axonal marker per 100 μm axons) under control (0.86±0.06) or after nicotine application (1.65± 0.08, **P< 0.01, One-Way ANOVA Post Hoc Tests). KN93, a CaMKII inhibitor, had no effect on basal phospho-CaMKII immunofluorescent intensities (0.76± 0.02), but blocked nicotine activation of CaMKII (0.80± 0.04). Total CaMKII immunofluorescent intensities along axons were also quantified (as ratio of tCaMKII/pan-axonal marker per 100 μm axons) under control (1.08±0.19) or after nicotine application (1.12± 0.24, *P*> 0.05, One-Way ANOVA Post Hoc Tests). **C**: The time-course of CaMKII activation was quantified. vHipp axons were fixed in 1, 5, 15, 30, and 60 min respectively after nicotine treatment. phospho-CaMKII immunofluorescent intensities along vHipp axons increased quickly (1.65± 0.08 at 1’) and remained elevated for at least 30 min (1.75± 0.12 at 5’, 1.76± 0.09 at 15’, and 2.00± 0.10 at 30’, **P< 0.01, One-Way ANOVA Post Hoc Tests), returning to control levels at 60 min (0.84± 0.04). **D**: vHipp microslices culture from either WT or α7-/- mice were fixed 5 min after the incubation of nicotine (with 15’ pre-incubation of αBgTx or DHβE) or α7*nAChR selective agonist choline (10 mM for 1 min). αBgTx (100 nM), α7*nAChR selective antagonist, blocked (1.11± 0.08) the nicotine activation of CaMKII in vHipp axons, but DHβE (1 μM), the non-α7*nAChR selective antagonist, did not (1.60± 0.12). Choline (10 mM), the selective α7*nAChR agonist, activated (1.76± 0.04) axonal CaMKII. Neither nicotine (1μM, 0.88± 0.04) nor choline (10 mM, 0.86± 0.08) activated CaMKII in vHipp axons from α7-/- mice. **E**: vHipp microslice cultures from WT mice were fixed 5 min after nicotine treatment with 30 min pre-incubation of CdCl_2_, ryanodine or Xes-C. Pre-incubation with CdCl_2_ (100 μM, 1.61± 0.08), a voltage-gated calcium channel blocker, or ryanodine (30 μM, 1.62± 0.11), an intracellular calcium store ryanodine receptor blocker, did not affect nicotine activation of CaMKII in vHipp axons. Pre-incubation with Xes-C (100 nM, 0.90± 0.04), an intracellular calcium store IP_3_ receptor blocker, prevented nicotine activation of CaMKII. Data represent the mean ± SEM, **P< 0.01, One-Way ANOVA Post Hoc Tests. At least 1500 μm axonal lengths for each group from three independent experiments were collected and quantified.

The Ca^2+^/calmodulin-dependent autophosphorylation at Thr286 of CaMKII has been implicated in many aspects of pre- and post-synaptic regulation. We next tested whether the effects of nicotine on presynaptic Ca^2+^ might involve activation and autophosphorylation of CaMKII. WT vHipp slices were briefly exposed to low concentrations of nicotine (1 μM), fixed, and then examined for immunoreactivity with phospho-CaMKII *vs.* pan axonal marker antibodies. Phospho-CaMKII was detected at discrete sites along vHipp axons within 1 min of nicotine exposure. By quantifying the amount of phospho-CaMKII signal along vHipp axons at different time points after the removal of nicotine, a time course of nicotine activated CaMKII was obtained. Under these conditions, “hot spots” of autophosphorylated CaMKII were detected for ^~^30 min after nicotine washout with the signal returning to baseline by 60 min after nicotine washout (Figure **6A, B, C**). The focal activation of phospho-CaMKII by nicotine was blocked by pretreatment with the CaMKII inhibitor, KN93 (30 min, 5 μM) (Figure **6B**).

We also examined the total amount of CaMKII along vHipp axons by measuring total-CaMKII immunoreactivity (*vs.* pan axonal antibody staining): nicotine application did not affect the total amount of CaMKII along vHipp axons (Figure **6B**). These data are consistent with the idea that nicotine activates axonal phospho-CaMKII at multiple presynaptic locations. 

Using genetic and pharmacological methods, we further dissected the nAChR subtypes contributing to nicotine activation of CaMKII along vHipp axons. Pre-incubation (15 min) with the α7*nAChR selective antagonist (αBgTx, 100 nM) blocked the nicotine-induced increase of phospho-CaMKII along vHipp axons. DHβE (1 μM), a nAChR antagonist that does not block α7*nAChR, was without effect (Figure **6D**). Choline (10 mM), an α7*nAChR agonist also activated CaMKII along vHipp axons from WT mice. Finally, in studies of vHipp axons from α7-/- mice, neither nicotine nor choline increased CaMKII phosphorylation (Figure **6D**). Taken together, these results are consistent with the idea that the activation of α7*nAChR is both necessary and sufficient to activate axonal CaMKII.

As delineated above, IP_3_ receptor-mediated CICR contributes to the sustained phase of the α7*nAChR mediated change in [Ca^2+^]_i_ in vHipp axons. To further test whether CICR is required for α7*nAChR mediated activation of CaMKII, we treated vHipp microslices with ryanodine or xestospongin C for 30 minutes prior to nicotine application. Pre-incubation with xestospongin C (100 nM) blocked the nicotine-elicited increase of phospho-CaMKII whereas ryanodine (30 μM) treatment had no effect (Figure **6E**). Likewise, blockade of voltage-gated calcium channels by incubation with CdCl_2_ (100 μM) was without effect on nicotine activation of phospho-CaMKII. Thus, activation of α7*nAChR, and the subsequent IP_3_ receptor mediated CICR appear to be requisite steps in the nicotinic activation of CaMKII along vHipp axonal projections.

## Discussion

Nicotine activation of presynaptic nAChRs enhances neurotransmitter release, eliciting both short and long-term potentiating effects on synaptic plasticity [30,33,43,44]. Modulation of Ca^2+^ entry at presynaptic terminals is an important step in determining the extent of neurotransmitter release. We had proposed that nAChR-mediated stimulation of both Ca^2+^ influx and subsequent Ca^2+^ dependent signaling might be involved in the sustained phase of nicotine-induced facilitation of synaptic transmission [33]. In the present work, we investigated nicotine induced calcium signaling in presynaptic axons using *in vitro* microslices culture preparations. The most striking finding of the current study is that a brief exposure to nicotine elicits prolonged changes in Ca^2+^ signaling along axonal projections from ventral hippocampus. Because this result was so unexpected, we have attempted to probe the underlying mechanisms in the series of experiments documented here. 

**Figure 7:**
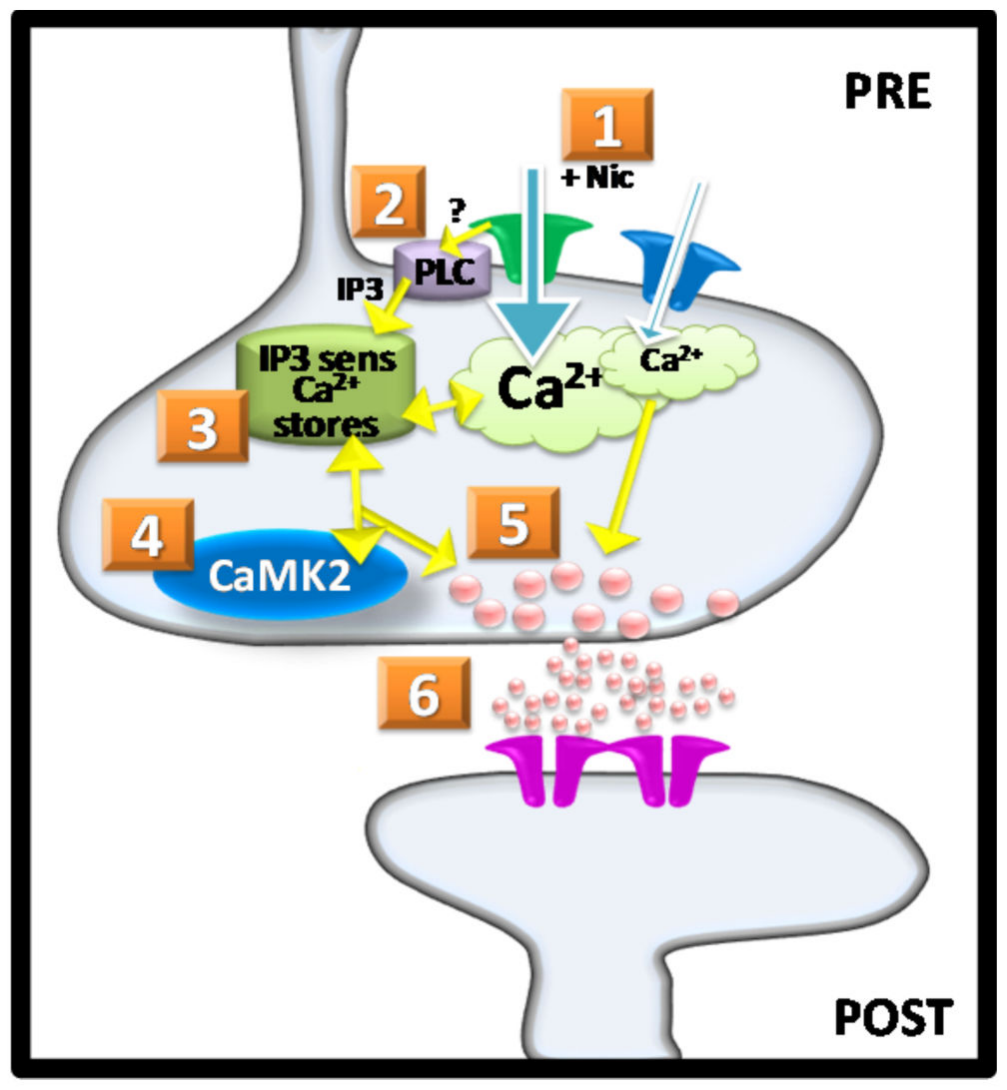
Model showing role of presynaptic α7*nAChR mediated calcium signaling in sustained facilitation of synaptic transmission. **Step 1**, Activation of presynaptic nAChRs by nicotine induces calcium influx into the presynaptic terminals. Activation of non-α7*nAChR (low Ca^2+^-permeability) elicits small, acute increases in intracellular Ca^2+^ at presynaptic terminals. **Step 2**, Activation of α7*nAChR (high Ca^2+^-permeability) elicits a larger increase in intracellular Ca^2+^ at presynaptic terminals which is sufficient to induce calcium release from the IP_3_-sensitive internal Ca^2+^ stores and maintain sustained changes in intracellular Ca^2+^; activation of α7*nAChR also can activate PLC by unknown mechanisms. PLC generates IP_3_ which mobilizes IP_3_ receptor controlled calcium stores (**Step 3**)**. Step4**, CaMKII is also activated by activation of α7*nAChR, and active CaMKII in turn, via a positive feedback mechanism, sustains the elevation of presynaptic Ca^2+^. **Step 5 and 6**, The sustained change in intracellular Ca^2+^ and activation of CaMKII at presynaptic terminals triggers the long-term enhancement of glutamate release, inducing long-term facilitation of glutamatergic synaptic transmission through glutamate receptors.

Figure **7** presents a working model of the proposed steps in signaling that may underlie the sustained phase of Ca^2+^ signaling and enhanced glutamate release at vHipp-nAcc synapses following nicotine exposure. The first step involves activation of nAChRs by nicotine (Figure **7** **, Step 1**). Activation of nAChRs can result in Ca^2+^ influx from direct permeation of the receptor channel as well as activation of voltage gated Ca^2+^channels. The calcium imaging data in the present study indicates that low concentrations of nicotine can lead directly to increases in [Ca^2+^]_i_ in presynaptic vHipp axons. We excluded many of the potential indirect effectors by blocking a) action potential-induced synaptic transmission (with TTX), b) NMDA-receptor (with AP-5), c) AMPA-receptor (with CNQX), d) mGluR (with LY341495), e) muscarinic ACh receptors (with atropine) and f) GABA-receptor (with bicuculline). Recently, several labs have shown that activating nAChRs in astrocytes induces Ca^2+^ signaling and modulates synaptic plasticity pre- or post-synaptically by the release of gliotransmitters, such as ATP [39,45,46]. An indirect effect of nicotine on astrocytic nAChRs is unlikely to account for the current findings for several reasons. First, the vHipp projections in vitro are devoid of GFAP expression and hence devoid of astrocytes. Second, the ATP receptor antagonist PPADS did not block the nicotine-induced calcium signaling along vHipp axons (data not shown). 

Our most surprising finding was the prolonged time course of the Ca^2+^ response following brief exposure to and then washout of nicotine. Although it has been reported that nicotine can induce calcium transients by activation of nAChRs in cultured neurons and in several cell types that express nAChRs [36–38], even the most prolonged time-courses recorded were in the seconds to minutes range. The lack of reports of more extensive Ca^2+^ signaling may be due to difficulties with performing long term recordings of intracellular Ca^2+^ signals due to photo damage of tissue. With the spinning-disc confocal, the CCD and rapid image collection used in the present study we were able to obtain more extended live calcium imaging. Using this configuration, we have routinely recorded Ca^2+^ signals for up to 1 hour without any apparent damage to the neurons.

The schematic in Figure **7** also indicates that α7*nAChR is specifically required for the sustained phase of nicotine-induced Ca^2+^ signaling along vHipp axons (Figure **7** **, Step 1**). Different subtypes of neuronal nAChR are differentially permeable to Ca^2+^, the α7*nAChR is the most permeable to Ca^2+^ [47,48]. Both α7* and non-α7*nAChR are present at presynaptic terminals and modulate neurotransmitter release and the strength of glutamatergic transmission [29-33]. We found that both α7* and non-α7*nAChR (including α4, Figure **1**; α5, and β2 subunits, data not shown) are present on the vHipp glutamatergic axons. Using genetic and pharmacological methods, we show that non-α7*nAChRs mediate a transient increase in [Ca^2+^]_i_, whereas activation of α7*nAChRs elicited a more sustained Ca^2+^ response along vHipp axons lasting ≥10-30 mins. Long lasting effects of brief nicotine exposure were also shown to require α7*nAChRs in studies of nicotine-induced long-term potentiation of synaptic plasticity [33,44,49]. 

Nicotine activation of α7*nAChR results in Ca^2+^ induced Ca^2+^ release (Figure **7** **, Step 2**). α7*nAChR have relatively short open-times and a fast rate of desensitization at high agonist concentrations [50,51]. Even at the very low concentrations of agonist used here, the Ca^2+^ influx via α7*nAChR is unlikely to be sufficient to maintain intracellular Ca^2+^ signals for the observed time course. Instead, we expect that activation of α7*nAChR initiates CICR from intracellular Ca^2+^ stores that participate in further intracellular Ca^2+^ signaling. Once the process is initiated α7*nAChR activation is no longer necessary; application of the α7*nAChRs antagonist αBgTx several minutes after nicotine washout had no effect on the subsequent sustained Ca^2+^ response (Figure **3**). 

Specific subtypes of nAChRs have been associated with defined Ca^2+^ signaling pathways: non-α7*nAChRs are mainly associated with Ca^2+^ signals mediated by activation of voltage dependent calcium channel (VDCC), whereas Ca^2+^ entry through the α7*nAChR channel itself can activate CICR from internal stores [21,52]. Previous studies have shown that in neuroblastoma cells and hippocampal astrocytes, α7*nAChR-mediated Ca^2+^ signaling primarily from CICR through ryanodine receptors [39,40,52]. In contrast, we found that in vHipp axons, the ryanodine receptor antagonist did not affect the sustained Ca^2+^ response to nicotine, but rather IP_3_ receptor-dependent Ca^2+^ stores were required for the α7*nAChR-mediated sustained Ca^2+^ response. It is still unclear how activation of α7*nAChR leads to increased IP_3_. However, recent studies in microglia, T cells and sperm have all demonstrated that α7*nAChR induced Ca^2+^ release is mediated by either src family kinases or PLC/IP3 [53–55]. Our studies demonstrated that PLC activity is required for nicotine induced CICR, via IP3 receptor controlled stores in vHipp axons. How α7*nAChRs stimulate PLC/IP3 signaling is not clear. Direct interactions between α7*nAChRs and a G-protein signaling complex have been reported in PC12 cells [56], raising the possibility that vHipp axonal α7*nAChRs also functionally interact with a G protein coupled signaling complex. 

M1 and M3 muscarinic acetylcholine receptors (mAChRs) are coupled to phospholipase C (PLC) via heterotrimeric G-protein (Gq/11), leading to increased IP_3_ and induced Ca^2+^ release from IP_3_-sensitive store [57]. A direct or indirect effect of nicotine on mAChRs is unlikely to account for the current findings because atropine, the competitive antagonist for the mAChRs, did not block the nicotine-induced calcium signaling along vHipp axons (data not shown).

Previous studies demonstrated that nicotine activates several second messenger cascades, involving protein kinase A [58], protein kinase C [59], phosphatidyl-inositol 3-kinase [60], MAPK [61] and CaMKII [62,63]. Of note, activated CaMKII has been associated with presynaptic neurotransmitter vesicles at synapses [64] and with the modulation of neurotransmitter release and synaptic transmission [65]. Nicotine treatment of vHipp axons locally activated CaMKII. We found that α7*nAChR mediated calcium signaling and the subsequent IP_3_ receptor mediated CICR appear to be requisite steps in the nicotinic activation of CaMKII at presynaptic sites, i.e. along vHipp axons. As the CaMKII inhibitor KN93 and AIP blocked the nicotine-induced sustained Ca^2+^response, we propose (Figure **7** **, step 3, 4**) that IP_3_ receptor-mediated CICR is both an activator of CaMKII and a key substrate of CaMKII that is involved in prolonged enhancement of neurotransmitter release at vHipp-nAccs synapses (Figure **7** **, step 5, 6**).

In conclusion, activation of presynaptic nAChRs by nicotine induces calcium influx into presynaptic axons. Activation of non-α7*nAChR (low Ca^2+^-permeability) elicits small and short-term Ca^2+^ signals along vHipp axons and thus induces a short-term facilitation of neurotransmitter release and synaptic transmission at vHipp-nAccs synapses [33]. By converting acute α7*nAChR activation into sustained cellular signaling, the increase in Ca^2+^ signals along presynaptic axons appears to be a crucial link between nAChRs and the downstream processes that participate in nicotine induced neurotransmitter release and potentiation of synaptic transmission [33].
